# Address before the Tennessee State Dental Association

**Published:** 1870-09

**Authors:** S. J. Cobb


### ARTICLE IV.

### *Address before the Tennessee State Dental Association.*

By S. J. Cobb. D. D. S.

Gentlemen of the Tennessee Dental Association.

Not having had an opportunity of returning my thanks at the time of your electing me President of our Society, I now thank you most kindly for the honor conferred. And while I am satisfied you could have selected another more competent to have served you, I will say I have discharged the duties of the office to the best of my ability, and therefore I hope you will excuse my many short comings and allow me to return thanks for courtesies extended during the meeting. This being my first effort on an occasion of this kind, I am sure you will not expect much in the way of a speech, in fact if I had the experience and ability to make you a long and interesting one, I do not think the occasion demands it, therefore I shall confine myself to a few remarks on the importance of legitimate associated effort. This gentlemen has ever been a subject of interest with me. I am aware of the fact that we have some few in our profession who are, to some extent, opposed to societies, and, I am sorry to say, a goodly number who have yet to manifest much interest in them. This, in my opinion, is attributable to two cause, in the first place our profession being young it has not had time to build up sufficiently to interest the masses in this way, and in the second place, as is the case in everything else, some few men have attempted to take hold and run some of our societies as they would machinery, for the purpose of grinding out their own agrandizement. In this way I have no doubt some have been brought to a premature end. This I trust will never be the case with our Tennessee Association. In the life and death of others I hope we have had experience enough to enable us (as doctors) to administer to the wants of this society, to make it not only long lived, but sufficiently healthy to ingraft itself upon the next generation as a permanent and indispensable institution. To do this it becomes the duty of every dentist in the State, to put his shoulder to the wheel and see that all push together—this is the object of associated effort; it is utterly impossible to accomplish singly and alone such results as can otherwise be done.

If any one doubts this statement let him cast his eye back over the history of the past, and behold how sublime the result of combined effort. It makes a garden of the wilderness, a civilized man of the heathen; by it our institutions of learning spring up and flourish, and our legislation is accomplished, and it is by united effort that our church organizations are enabled to wield such influence over the masses. By it governments are made and destroyed, in a word united effort wields the thunderbolt of power, it is the Archimedian lever by which the world is moved.

This being true, why should we hesitate to organize, drill and discipline our forces for the great victory to be achieved by associated effort.

We have at present about two hundred dentists in our State, the same number going out to do battle for their country, would organize, drill and discipline themselves in such a way as not only to make every move scientifically, but jointly and understandingly. If this be necessary for men who go into the field to kill one another, I am sure it is none the less so for us, as a little dental army, to do battle for the living.

It is true our enemy does not come with sword and bayonet in hand, but so disguised and in so many different ways, that it requires ten times the science and skill to defeat, as does the bold and daring soldier. As such I appeal to every gentleman present to look well to the interest of this society, to see that each and every one returns next year not only a better dentist, but with a number of new and good applicants for membership. Let us go on in this way until we get into our ranks every respectable dental practitioner in the State, and as many more as may wish to join. In the mean time we must see that nothing is left undone that will contribute to our science, skill, and brotherly love. I fear gentlemen we do not understand and appreciate the importance of brotherly love. To advance in dental science as we are capable of doing, we should work as a band of brothers. I do not see why we should not to a very great extent become personal friends, especially where we reside in the same cities and towns; in this way we might put an end to our professional bickerings and jealousies; the aim and end of all in one sense are the same. We are all engaged in the practice of dentistry for the purpose of making money, or in other words a living; the great misfortune is, we do not understand ourselves, one will go at it in one way and another in another, some take so little thought upon the subject that they conclude it is best to humbug and swindle everybody they come in contact with, while others seem to think they can do nothing except at the expense of others; as a matter of course they are always trying to pull others down to get up themselves. Then again we find others who are so jealous and narrow minded, that they can see no room upwards for any one but themselves. This gentlemen is all wrong, the platform of greatness is sufficiently large for the whole human family; as for pulling others down to get up ourselves, it is simply a mistake for the want of better judgement, and when it comes to cheating and defrauding, it is certainly a disgrace to all who are engaged in it, while this little narrow minded sickly view, prompted by jealousy, is little better. To sum the whole thing up, it seems to be attributable to the want of more knowledge of the great living principle that rules and governs all matter, man included. This great principle establishes the fact that what is good for the whole is equally good for an individual. No one who comprehends and thoroughly understands it, can afford to engage in humbugging his fellow man, or attempt to elevate himself upon the downfall of others; neither will he in any way appropriate the labor of others, nor say or do any thing whatever that will hinder any one from reaching the top-most round in the ladder. He knows too well he is a part and parcel of the same; he understands enough of the philosophy of life to know that the happiness of man depends upon the conduct of one another, it is an established fact with him that no one can live within himself. To obtain the greatest degree of excellence and happiness he must endeavor to take all upon a level with himself, onward and upward. I hope every member of this Society will take up this subject, and investigate it thoroughly, get the truth out of it, and govern himself accordingly, just as we do this we become better men, do better dentistry, make more money, live longer, and enjoy life the better. In conclusion, gentlemen, I hope you will allow me to call attention to our constitution, by laws, and ethics, which you will find on the Secretary's table. Those who have not provided themselves with a copy, I hope will do so before leaving, and when you go home read carefully, so as to understand thoroughly, and live up to it to a letter. We do not claim for it perfection, but it is the best we have thus far been able to get up ; by learning and living up to it, we will be the better able to make good its imperfections. Some may argue, which I have heard even in our American Dental Association, that we need nothing of the kind, but when we take into consideration that law is the great civilizer of man, and without it, we would simply be a set of desperadoes, constantly engaged iu pillage and murder, it is evident that we can not get along without it. Without law our happiness and comfort in life would be limited to a very great extent; it is intended as the sign board at the forks and crossings of roads, to direct us in the way we should go. The man who says he will dispense with it, had as well say he can travel successfully the world over without reading a sign or asking a question in regard to his rout. Our constitution, by laws and code of ethics, are extended to direct us in the way we should go, and I for one insist on every member of this society going on as it directs, and if any part of it should lead us astray, we will find it out and correct the error; in this way we may become as a band of brothers laboring as one man.

